# Prevalence and Risk Factors of Paternal Postpartum Depression in Multiple Primary Healthcare Centers in Saudi Arabia: A Cross-Sectional Study

**DOI:** 10.7759/cureus.80302

**Published:** 2025-03-09

**Authors:** Lujain Bokhari, Areej Alsulami, Ghaida Alharbi, Reem Nughays, Rozan Alabdali, Samiyah Alharthi, Sultan Magliah, Ahmad Alsabban, Rania Zahid, Abeer Abduljabbar

**Affiliations:** 1 College of Medicine, King Saud Bin Abdulaziz University for Health Sciences, Jeddah, SAU; 2 Department of Family Medicine, Ministry of the National Guard-Health Affairs, King Abdulaziz Medical City, Jeddah, SAU; 3 Department of Family Medicine, Ministry of National Guard Health Affairs (MNG-HA) King Abdulaziz Medical City, Jeddah, SAU; 4 Department of Family Medicine, King Abdullah International Medical Research Center, Riyadh, SAU

**Keywords:** edinburgh postnatal depression scale, paternal postnatal depression, paternal postpartum depression, risk factors, saudi arabia

## Abstract

Introduction

Paternal postpartum depression (PPPD) is an important public health issue that can negatively affect relationships among fathers, partners, and children. However, research on this issue in Saudi Arabia is lacking. We aimed to determine the prevalence and risk factors for PPPD among Saudi fathers.

Methods

This cross-sectional study was conducted among Saudi fathers in King Abdulaziz Medical City, Jeddah. We included all fathers of newborns aged ≤6 months while excluding those with a history of depressive disorder. A self-administered questionnaire was sent to all participants. The Arabic version of the Edinburgh Postnatal Depression Scale (EPDS) was used to screen individuals for PPPD.

Results

The total number of respondents was 223. Approximately 30.9% of participants had PPPD based on an EPDS cutoff value of ≥9. Significant risk factors associated with PPPD were whether the father had lost a child (p = 0.0083) and whether the father had a family history of depression (p = 0.0028).

Conclusion

Our findings suggest that PPPD is prevalent among Saudi fathers; therefore, there is an urgent need for further research on PPPD. The results of this study will contribute to establishing preventive and intervention programs.

## Introduction

Depression is a common mental disorder affecting approximately 280 million people, 5% of whom are adults. Depression also contributes considerably to the global burden of disease and was ranked as the second most disabling illness by 2020 [1-3]. The etiology of depression varies according to genetic and environmental factors, and diagnosis is based on depressive symptoms experienced during the past two weeks. This must include loss of interest in daily entertaining activities or a depressed mood, and four or more of the following: fatigue, sleep disturbances, concentration difficulties, changes in appetite or weight, negative thoughts, psychomotor abnormalities, or suicidal ideation [1,4]. Usually, mild depression can be treated with psychological interventions such as cognitive behavioral therapy (CBT), while moderate-to-severe depression can be treated with a combination of CBT and pharmacological therapy such as antidepressant medications [5].

Postpartum depression (PPD) mainly affects women a month after childbirth, with an incidence of 10-15% [4]. According to the Diagnostic and Statistical Manual of Mental Disorders, Fifth Edition, the diagnosis of PPD must include the criteria for major depressive disorder and a peripartum onset specifier [4]. The definition of paternal PPD (PPPD) resembles that of maternal PPD, wherein fathers experience major depressive episodes within the first month of childbirth, and these episodes include one of the clinical manifestations of depression [6].

To diagnose women with PPD, the Edinburgh Postnatal Depression Scale (EPDS), a diagnostic tool composed of 10 self-rating questions assessing the patient’s psychological symptoms, has been used to evaluate depression severity [7]. The EPDS is also a valid tool for diagnosing men with PPPD [8]. The risk factors for PPPD can be divided into biological and environmental. Biological risk factors include changes in levels of various hormones, such as testosterone, estrogen, cortisol, vasopressin, and prolactin, during or after pregnancy [9-12]. Some studies have demonstrated that the relationship between depression and hormonal level changes is such that fathers usually become more aggressive, stressed, and less emotionally attached to their infants because of these hormonal changes [12-17]. Regarding environmental risk factors, excessive stress from becoming a parent, lack of social support, feeling neglected by their partner due to mother-infant attachment, and maternal PPD can all contribute to the development of PPPD [18-21]. The consequences thereof on fathers’ lives include failure to develop a secure attachment with the infant, an increase in marital issues, and a predisposition of the partner to maternal PPD [22-24].

A systematic review and meta-analysis in 2010 reported that men were less likely to develop depression than women in Saudi Arabia, with a relative risk of 0.65 (95% CI (54%, 80%)) [25]. Furthermore, a systematic review and meta-analysis conducted in 2022 measured the prevalence of mental health problems, including depression, and identified risk factors among the Saudi Arabian population during the coronavirus pandemic in 2019. The prevalence of depression was 30% (95% CI (22%, 38%)), and the risk factors associated with depression were divided into three categories: sociodemographic factors, such as female sex and poor income level; behavioral and health status-related factors, such as smoking and poor immune status; and COVID-19-related factors, such as lack of information about the virus and confirmed infection of a family member [26].

Over the past five decades, researchers from different countries have investigated maternal PPD more than PPPD, as it is a newly introduced phenomenon, and only three cross-sectional studies have been conducted in Saudi Arabia between 2019 and 2020 [27-30]. The aims of these studies were to evaluate the prevalence and identify the risk factors of PPPD among fathers of 4-8-month-old, 6-month-old, and 12-month-old newborns, with sample sizes of 150, 290, and 226 participants, respectively [28-30]. The prevalence of PPPD among Saudi fathers was 27.3%, 16.6%, and 32.7%, respectively [28-30]. Moreover, one of the three studies identified a significant risk factor, where being emotionally isolated from their wife was strongly associated with PPPD, with a p-value of 0.013 [29].

Men have been reported to seek mental health treatment at a rate that is roughly 50% lower than that of women, and the expenses for services were higher for fathers with paternal perinatal depression than for those without depression [31-32].

Because fathers also experience depression, and studies on this demographic are lacking, there is an urgent need for more research on PPPD, as it is not a well-recognized phenomenon. We, therefore, aimed to measure the prevalence of PPPD among fathers in Saudi Arabia and to determine the associated factors.

## Materials and methods

Study design, setting, and participants

This cross-sectional study was conducted among fathers of ≤6-month-old infants in King Abdulaziz Medical City (KAMC) in Jeddah, Saudi Arabia. KAMC operates five hospitals located in Riyadh, Jeddah, Al-Hassa, Dammam, and Al-Madinah, providing high-quality primary, secondary, tertiary, and quaternary healthcare services to Saudi Arabian National Guard personnel, civilians, employees, and their dependents. The KAMC in Jeddah, which has a bed capacity of 751, is a tertiary healthcare center delivering medical care services to the population in the Western Region. The study was conducted at the baby vaccine clinic of King Saud bin Abdulaziz University and the well-baby clinics of three primary healthcare (PHC) centers operated by KAMC in Jeddah: Iskan PHC, Bahra PHC, and Specialized Polyclinic PHC. We included all fathers of ≤6-month-old newborns who attended these clinics from January 1, 2022, to January 27, 2023, and excluded all fathers with a history of depressive disorder. Using the BestCare program (electronic medical records), all clinic visitors who met the study’s inclusion criteria were selected, and their contact numbers were obtained to further investigate the exclusion criterion. We contacted the participants via phone call and sent the questionnaire through SMS to those who did not answer, and resent it when they did not reply to the message.

Instrument

The main study tool was a self-administered questionnaire comprising 41 questions in two parts. The first part was designed to gather patient demographics, wife-, pregnancy and delivery-, infant-, family-, and father’s emotional status-related information. To assess the readability of this part of the questionnaire, it was sent randomly to eight fathers of ≤6-month-old newborns to obtain feedback, and modifications were applied to the survey accordingly. The tool was also face validated by two experts in the research field, psychologists, and family medicine consultants.

The second part of the questionnaire was the EPDS, which is composed of 10 self-rated questions originally designed to assess the severity of maternal PPD and is also a valid tool for diagnosing PPPD [7,8]. Moreover, a translated Arabic version of the EPDS was used to screen patients, as the native language of most of the participants was Arabic [33]. Each question in the EPDS has four choices, with scores ranging from zero to three. The maximum and minimum scores were 30 and zero, respectively. Depressive symptoms were identified using the scale during the 7 days prior to the interview.

Variables

For a better understanding of the relationship between the development of PPPD and other factors, the first part of the questionnaire included questions about the following factors: (1) participants’ demographics, including father’s age, educational level, employment status, profession, income, overall health status, current medications, psychological disorders, and sexual dysfunction disorders; (2) information related to the wife, including marital status, number of wives, employment status, and overall general health; (3) information related to the pregnancy and delivery, including the mode of delivery, complications during pregnancy or labor, whether the pregnancy was planned, whether the father attended the delivery, whether the father has ever lost a child before, and whether the wife stayed at her family’s home after delivery; (4) information related to the infant, including sex, age, overall general health, number of children, and whether the baby was the father’s first child; (5) family information, such as type of family structure, the relationship between the father and his family, the relationship between the father and his wife’s family, and whether there were any close relatives diagnosed with depression; and (6) information related to the father’s emotional status, which included questions about whether the father was emotionally disconnected from his wife or his child.

Statistical analysis

The collected data were entered into JMP Pro software (John’s Macintosh Project), version 10.0 (SAS Institute Inc., Cary, NC, USA) for statistical analysis. Qualitative variables such as the father’s educational level, father’s marital status, mode of delivery, infant’s sex, and family structure type are presented as frequencies and percentages, and quantitative variables such as father’s age and number of children are presented as medians and IQRs. The chi-square and Fisher’s exact tests were used to compare qualitative variables between fathers with or without depression, whereas the Wilcoxon rank-sum test was used to compare quantitative variables. Statistical significance was set at p < 0.05.

To calculate the prevalence of PPPD, the number of fathers with an EPDS cutoff value of ≥9 was divided by the total number of fathers included in the study. Multiple logistic regression analysis was used to determine the risk factors for PPPD, and the results are expressed as p-values, ORs, and 95% CIs.

Ethics approval and consent to participate

This study was approved by the King Abdullah International Medical Research Center Institutional Review Board (approval number: SP22J/012/02). The purpose of the study, survey duration, and participant anonymity were explained to each participant. Verbal consent was obtained from the participants to ensure volunteerism. A copy of the informed consent form was included with the online survey.

## Results

The questionnaire was sent to 448 fathers who met the study’s inclusion criteria, 238 of whom responded. Fifteen were excluded because they missed some survey questions or had a history of depression. Therefore, 223 participants were included, and the response rate was 53.13%.

Demographic variables

The median age of all respondents was 34 years (IQR = 9 years). Of the 223 men, 38 (17.04%) were aged ≥25 years, 132 (59.19%) were ≥30 years, 46 (20.63%) were ≥40 years, and 7 (3.14%) were ≥50 years. Regarding education levels, approximately half of the participants, 121 (54.26%), stated they had a higher education certificate. Almost all fathers, 206 (92.38%), reported that they were employed, 190 (85.20%) of whom were employed in the military sector. The monthly salary of 114 (51.12%) participants was < 10,000 Saudi Riyals. Nearly all participants, 185 (82.96%), were disease-free; 25/38 of the remaining fathers with chronic diseases mentioned the medications they were currently using, which included nine participants taking anti-hyperglycemic medications (insulin); five, antihypertensive medications; five, cholesterol-lowering medications; two, rheumatism medications; two, asthma medications; one, aspirin; and one participant using a proton pump inhibitor. Additionally, 219 (98.21%) and 220 (98.65%) fathers confirmed they did not have psychological or sexual disorders, respectively. No significant associations were observed between demographic variables and depression when comparing fathers with and without depression (Table 1).

**Table 1:** Demographic data of fathers with/without depression. *P-value is based on the Wilcoxon rank-sum test. ^P-value is based on the chi-square test. ^^P-value is based on Fisher's exact test. IQR = Interquartile Range; SR = Saudi Riyals.

Variables		Overall n = 223	Depression n = 69	No depression n = 154	p-value
Age in years, median (IQR)		34 (9)	33 (8)	35 (10)	0.250*
Educational level, n (%)	Received higher education	121 (54.3)	37 (30.6)	84 (69.4)	0.898^
	Did not receive higher education	102 (45.7)	32 (31.4)	70 (68.6)	
Employment status, n (%)	Employed	206 (92.4)	63 (30.6)	143 (69.4)	0.686^
	Unemployed	17 (7.6)	6 (35.3)	11 (64.7)	
Profession, n (%)	Military sector	190 (85.2)	60 (31.6)	130 (68.4)	0.551^^
	Private civil sector	7 (3.1)	1 (14.3)	6 (85.7)	
	Public civil sector	20 (9.0)	5 (25)	15 (75)	
	Unemployed	6 (2.7)	3 (50)	3 (50)	
Monthly income in SR, n (%)	<10000	114 (51.1)	41 (36.0)	73 (64.0)	0.229^
	10000–15000	78 (35.0)	21 (27.0)	57 (73.1)	
	>15000	31 (13.9)	7 (22.6)	24 (77.4)	
Medically healthy, n (%)	Yes	185 (83.0)	55 (29.7)	130 (70.3)	0.388^
	No	38 (17.0)	14 (36.8)	24 (63.2)	
Any psychological disorders, n (%)	Yes	4 (1.8)	2 (50)	2 (50)	0.589^^
	No	219 (98.2)	67 (30.6)	152 (69.4)	
Any sexual dysfunction disorders, n (%)	Yes	3 (1.4)	1 (33.3)	2 (66.7)	1.000^^
	No	220 (98.7)	68 (30.9)	152 (69.1)	

Wife, pregnancy and delivery, infant, family, and father’s emotional status variables

All 223 (100%) respondents were married. Almost all fathers had one wife, accounting for 211 (94.61%) participants. Most men, 200 (89.69%), had unemployed wives. Furthermore, 198 (88.79%) wives were medically healthy.

Concerning fathers’ feelings about pregnancy, most fathers, 191 (85.65%), confirmed the pregnancy was planned. Additionally, 151 (67.71%) wives did not experience complications during pregnancy or delivery. In total, 136 (60.99%) participants did not attend labor. Most fathers, 156 (69.96%), reported that the child was delivered through vaginal delivery, and 117 (52.47%) reported that their wives stayed at their family home after delivery. Forty-three (19.28%) fathers had lost children previously.

Of the participants, 159 (71.3%) reported being experienced fathers. Almost half of the infants were male, representing 122 (54.7%) infants, and three (1.35%) were identical twins. Moreover, 130 (58.3%) infants were >4 months of age. The median number of children in each family was two (IQR = 3). Regarding infant health status, 206 (92.38%) infants were medically healthy.

Approximately three-quarters of the fathers, 168 (75.34%), belonged to nuclear families. Almost all fathers, 220 (98.65%), have good relationships with their parents and their families-in-law. Only 28 (12.56%) respondents have close relatives who were diagnosed with depression.

Concerning the emotional status of fathers, 211 (94.62%) reported no emotional disconnection from their wives, and 221 (99.10%) reported no disconnection from their infants. No significant associations were observed between these emotional variables and paternal postpartum depression when comparing fathers with and without depression. However, three variables demonstrated statistically significant differences between these groups: previous loss of a child (p = 0.005, test value = 7.984, 95% CI (19.2, 75.4)), medical health of the child (p = 0.002, test value = 9.818, 95% CI (165.2, 1323.6)), and family history of depression among close relatives (p = 0.001, test value = 10.287, 95% CI (12.4, 63.1)) (Table 2).

**Table 2:** Information related to the wife, pregnancy and delivery, infant, family, and father’s emotional status. *P-value is based on the Wilcoxon rank-sum test. ^P-value is based on the chi-square test. ^^P-value is based on Fisher’s exact test.

Variables		Overall n = 223	Depression n = 69	No depression n = 154	p-value
Information related to the wife					
Number of wives, n (%)	One	211 (94.6)	64 (30.3)	147 (69.7)	0.409^
	More than one	12 (5.4)	5 (41.7)	7 (58.33)	
Wife’s employment status, n (%)	Employed	23 (10.3)	7 (30.4)	16 (69.6)	0.956^
	Not employed	200 (89.7)	62 (31)	138 (69)	
Medically healthy wife, n (%)	Yes	198 (88.8)	60 (30.3)	138 (69.7)	0.562^
	No	25 (11.2)	9 (36)	16 (64)	
Information related to the pregnancy and delivery					
Father’s feelings about this pregnancy, n (%)	“I wanted this pregnancy”	191 (85.7)	55 (28.8)	136 (71.2)	0.207^^
	“I do not know”	22 (9.9)	10 (45.5)	12 (54.6)	
	“I did not want this pregnancy”	10 (4.5)	4 (40)	6 (60)	
Any complications during pregnancy or delivery, n (%)	Yes	72 (32.3)	24 (33.3)	48 (66.7)	0.594^
	No	151 (67.7)	45 (29.8)	106 (70.2)	
Attended the delivery, n (%)	Yes	87 (39)	27 (31)	60 (69)	0.981^
	No	136 (61)	42 (30.9)	94 (69.1)	
Delivery mode, n (%)	Normal delivery	156 (70)	50 (32)	106 (68)	0.584^
	Cesarean delivery	67 (30)	19 (28.4)	48 (71.6)	
Wife stayed at her family’s home after delivery, n (%)	Yes	117 (52.5)	42 (35.9)	75 (64.1)	0.093^
	No	106 (47.5)	27 (25.5)	79 (74.4)	
Lost a child before, n (%)	Yes	43 (19.3)	21 (48.8)	22 (51.2)	0.005^
	No	180 (80.7)	48 (26.7)	132 (73.3)	
Information related to the infant					
First child, n (%)	Yes	64 (28.7)	21 (32.8)	43 (67.2)	0.701^
	No	159 (71.3)	48 (30.2)	111 (69.8)	
Sex, n (%)	Female	98 (44)	29 (29.6)	69 (70.4)	0.388^^
	Male	122 (54.7)	38 (31.2)	84 (68.9)	
	Twins of the same sex	3 (1.4)	2 (66.7)	1 (33.3)	
Child’s age in months, n (%)	4 months or less	93 (41.7)	26 (28)	67 (72)	0.415^
	More than 4 months	130 (58.3)	43 (33.1)	87 (66.9)	
Children number, median (IQR)		2 (3)	2 (2)	2 (3)	0.421*
Medically healthy child, n (%)	Yes	206 (92.4)	58 (28.2)	148 (71.8)	0.002^
	No	17 (7.6)	11 (64.7)	6 (35.3)	
Information related to the family					
Family structure, n (%)	Extended family	55 (24.7)	16 (29.1)	39 (70.9)	0.732^
	Nuclear family	168 (75.3)	53 (31.6)	115 (68.5)	
Good relationship with parents, n (%)	Yes	220 (98.7)	68 (30.9)	152 (69.1)	1.000^^
	No	3 (1.4)	1 (33.3)	2 (66.7)	
Good relationship with wife’s family, n (%)	Yes	220 (98.7)	68 (30.9)	152 (69.1)	1.000^^
	No	3 (1.4)	1 (33.3)	2 (66.7)	
Any close relatives with depression, n (%)	Yes	28 (12.6)	16 (57.1)	12 (42.9)	0.001^
	No	195 (87.4)	53 (27.8)	142 (72.8)	
Information related to the father’s emotional status					
Emotionally disconnected from wife, n (%)	Yes	12 (5.4)	6 (50)	6 (50)	0.142^
	No	211 (94.6)	63 (29.9)	148 (70.1)	
Emotionally disconnected from child, n (%)	Yes	2 (0.9)	1 (50)	1 (50)	0.524^^
	No	221 (99.1)	68 (30.8)	153 (69.2)	

Prevalence of PPPD

**Figure 1:**
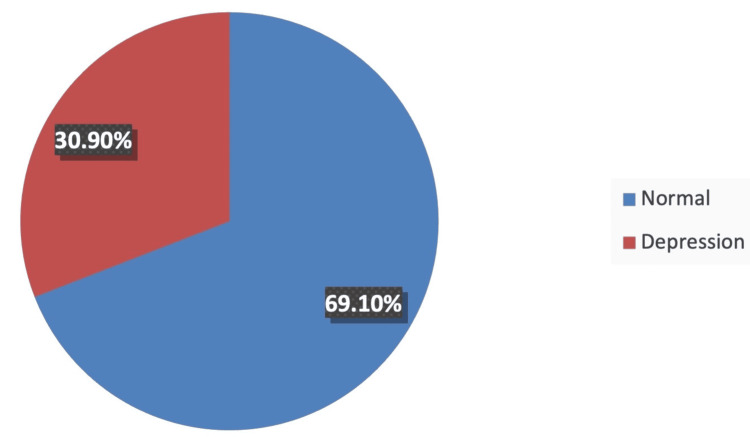
Prevalence of PPPD in Saudi Arabia. PPPD: Paternal postpartum depression.

Sixty-nine (30.9%) of the included participants had depression, as they scored ≥9 on the EPDS, which is the validated cutoff value indicating the probability of having depression. It has a sensitivity of 77.8% and a specificity of 81.3% (Figure 1) [29].

Risk factors for PPPD

To determine the risk factors for PPPD, multiple logistic regression analysis was performed with maximum likelihood estimation to obtain the OR. Predictors were selected based on a significant relationship pattern with the outcomes. The initial model included the infant’s health status, whether the father had lost a child, and whether any close relatives were diagnosed with depression. Fathers who had previously lost a child and those who had close relatives diagnosed with depression were more likely to develop depression than fathers without these factors (Table 3).

**Table 3:** Risk factors for paternal postpartum depression. *P-value is derived from multiple logistic regression analysis.

Variable	OR	95% CI	p-value
Previous loss of a child (Yes vs. No)	2.58	(1.28-5.22)	0.0083*
Family history of depression (Yes vs. No)	3.52	(1.54-8.19)	0.0028*

## Discussion

During the last five decades, maternal PPD has become more familiar than PPPD. If PPPD is not detected and proper medical intervention is not provided, it may lead to serious issues that impede fathers' personal, social, and occupational functions. We, therefore, aimed to calculate the prevalence of PPPD and investigate the possible factors that predispose fathers to this condition.

The measured prevalence of PPPD in this study, supported by the utilization of the EPDS with a cut-off value of nine, was 30.9%, indicating that one-third of all respondents experienced depression. This prevalence was relatively higher than that reported in previous studies conducted in Pakistan, China, France, Japan, and Iran, with prevalence rates of 23.5%, 7.5%, 5.7%, 11.2% at one month and 12% at six months, and 15.7%, respectively [34-38]. Furthermore, two studies in Australia and Taiwan showed results similar to ours, with prevalence rates of 30.5% and 31.3%, respectively [39,40]. Regarding Middle Eastern studies, the prevalence rate in Egypt was 31.8%, which is somewhat comparable to that of our study [41]. In Saudi Arabia, compared with our results, two studies conducted in Jeddah and Riyadh reported lower prevalence rates of 27.3% and 16.6%, respectively [28,29]. The Riyadh study reported an initial prevalence rate of 27.9%, but this was reduced to 16.6% based on tool sensitivity and specificity [29]. Another study in Riyadh reported a slightly higher prevalence (32.7%) of fathers with PPPD than in our study [30].

Specific differences exist between this study and previous studies. First, the timing of the depression assessment differed. For example, in Pakistan, the evaluation of PPPD was performed between 10 weeks and one year after childbirth, whereas in Riyadh, the assessment period was one year or earlier after delivery [29,34]. However, the participants in a study performed in Iran were fathers to newborns aged two to three days, while a study was conducted antenatally in Egypt [38,41]. Second, the cutoff value of the EPDS varied among studies. Regardless of whether it was validated, the cutoff value used in a Japanese study was ≥8, but in a Chinese study, it was ≥13 [35,37]. The studies conducted in Egypt and Jeddah applied a cutoff value of ≥10, but this was also not validated [28,41]. Third, some studies did not include fathers and mothers to assess PPD, such as that performed in France [36]. An explanation for the variance in prevalence rates between the present study and other studies is the difference in study designs, techniques, settings, EPDS cutoff rates, and postpartum screening tools. The period of depression evaluation, participant characteristics, sample size, and social and cultural diversity might have contributed to the divergence in the prevalence rates.

No relationship was found between employment status and PPPD, which is similar to the findings of a study conducted in Pakistan [34]. In contrast, two studies conducted in Japan and Nigeria found a significant correlation between unemployment status and PPPD [42,43]. Moreover, fathers’ educational levels were not statistically significant, which is consistent with a study performed in Saudi Arabia [30]. Two studies in the United Kingdom and Sweden revealed the opposite, in which there was an association between low educational level and depressive symptoms [44,45]. The age of the participants did not correlate with depressive disorder in our study, as opposed to earlier studies stating that the risk of PPPD is higher in younger fathers [45]. Based on our findings, fathers’ monthly income levels were not related to PPPD; although, according to studies conducted in Ireland and those in the United States with samples of Mexican American fathers, PPPD was significantly correlated with poor income [46,47]. A study conducted among Jamaican fathers revealed a negative correlation between high-income levels and the incidence of PPPD [48]. We found no relationship between PPPD and the father’s health status, the presence of psychological disorders, or the presence of sexual dysfunction disorders, which is comparable to a study conducted in Saudi Arabia [29]. Nevertheless, Peker AG et al. (2016) discovered that a history of psychiatric illness is a significant risk factor for PPPD [49]. Studies conducted in Ireland and Japan reported that a history of depressive disorder was significantly associated with PPPD [46,50]. However, we excluded fathers with histories of depression from our study. Regarding fathers’ health status, Underwood L et al. (2017) revealed a positive relationship between PPPD and poor health [51].

To our knowledge, the present study is the first to identify a significant association between fathers who have lost a child and experiencing PPPD. In contrast, a study in Canada reported no significant difference between perinatal loss in previous pregnancies and postnatal depression among fathers, similar to findings in Saudi Arabia and Egypt [29,41,52]. Additionally, maternal PPD has been reported as a significant risk factor for PPPD in studies conducted in Japan [37,53]. However, we did not investigate the relationship between maternal and paternal PPD. Similar to an Egyptian study, we found no relationship between unplanned pregnancy and PPPD, unlike previous studies that reported a correlation between unintended pregnancy and PPPD development [35,41,42,54,55]. Marital status was also not a significant factor in our study, though a study in Ireland found that being unmarried was significantly correlated with PPPD [46].

Moreover, several factors were not found to be significant in our study and preceding ones, such as the number of wives, wife's employment status, complications during pregnancy or delivery, the wife’s health status, fathers attending the delivery, and whether the wife stayed in her family’s home after delivery [28-30,41,43]. Furthermore, the mode of delivery was not significantly correlated with PPPD, comparable to another study in Saudi Arabia. However, a study in Stockholm, Sweden, found an association between fathers with depression and the mode of delivery [29,56].

Being a first-time father was not significantly associated with PPD in this study, similar to findings in Egypt, Saudi Arabia, and Japan [29,41,42]. We found that the child’s sex showed no correlation with PPPD, similar to a study in Jeddah [28]. However, a review study in Iran revealed that male sex showed a strong correlation in South Asia, East Asia, and Africa [57], and a Chinese study confirmed that the preference for male sex in infants is significantly related to PPPD development [58]. A child’s age, number of children, and health status showed no significance in this study, consistent with studies in Saudi Arabia [29,30]. Nonetheless, a negative correlation was observed between PPPD and the number of children in studies by Abdollahi F et al. (2021) and Roubinov DS et al. (2014), whereas Carlberg M et al. (2018) and Peker AG et al. (2016) reported the opposite [38,47,49,59]. Additionally, fathers of diseased infants under medical treatment were prone to developing PPPD, as reported in a Japanese study [37].

Family structure was not significant in this study, consistent with a study in Turkey [60]. However, Peker AG et al. (2016) found a positive correlation between the number of people living in the same house and EPDS scores [49]. The relationship between fathers and their parents and their wives’ families did not correlate with PPPD development, similar to a study in Riyadh [30]. Conversely, a Chinese study indicated a significant relationship between work-family conflict and PPPD development [61]. Surprisingly, our study detected a statistically significant relationship between PPPD and whether the father had a family history of depression, marking the first discovery of such a correlation. Conversely, although Shaheen NA et al., Ramchandani PG et al., and Fentz HNinvestigated whether having a family member diagnosed with depression increased the risk of developing PPPD, they did not find this to be statistically significant [29,62,63].

Regarding the father’s emotional status, being emotionally distant from the child was not related to PPPD, which corresponds with a study conducted in Riyadh [29]. Moreover, being emotionally distant from the wife was not a statistically significant factor for PPPD in our study, aligning with findings from another study conducted in Saudi Arabia. However, this contrasts with most previous research, where emotional distance from the wife was found to be significantly associated with PPPD [29,30,42,44,47,49,64].

This study had some limitations. First, the sample was small and was obtained from KAMC, Jeddah. Therefore, our findings cannot be generalized to the entire Saudi Arabian population [65]. However, the sample size was close to the average reported across studies, which was 313 [29]. Moreover, using a self-reported questionnaire increased the risk of recall bias because it required participants to recall past information, and social desirability bias, because the questionnaire included some private questions [66]. Furthermore, the response rate was 53.13%, which is low despite our efforts to increase it. The effect of maternal PPD on the participants was not clear because the EPDS was not applied to mothers. To address this issue, a self-reported questionnaire about the mother’s depression status should be sent to mothers separately, as the importance of this factor has been highlighted in previous studies. Additionally, it was not clear if the fathers’ relatives who were reported to have depression were actually medically diagnosed with depression or if the fathers merely suspected depression, which could affect the accuracy of the results.

Future prospective cohort studies at different time intervals during the perinatal period, involving multiple healthcare centers, are recommended to better understand the causality of PPPD. Both parents should be evaluated simultaneously to determine the relationship between maternal PPD and PPPD, which can be achieved by sending surveys to both parents. Personal interviews with psychologists are preferred in future studies, as these interviews can confirm the findings of the EPDS and reveal important risk factors for PPPD. Lastly, extending the postpartum vacation period in Saudi Arabia for fathers is recommended, which is currently only three days following childbirth. Philpott LF and Corcoran P (2018) have indicated that the absence of paternity leave can increase the risk of developing PPPD [46].

## Conclusions

The prevalence rate of PPPD among Saudi fathers was 30.9%, underscoring the need for involving fathers in the evaluation of perinatal mental health alongside their partners and establishing screening tools for PPPD. Given that PPPD is not widely recognized among healthcare workers, there is a high risk of missing diagnoses of depression in fathers. Therefore, it is advisable to educate professionals working in the field of perinatal medicine about PPPD. Notably, we identified significant risk factors for PPPD, which can inform the development of targeted prevention and intervention programs.
